# External Validation of Prognostic Models for Nonmetastatic Clear Cell Renal Cell Carcinoma in a Japanese Cohort: Evaluation of Predictive Performance

**DOI:** 10.1111/iju.70579

**Published:** 2026-07-23

**Authors:** Yoshinori Nakano, Shunsuke Miyamoto, Keisuke Goto, Kazuma Yukihiro, Shinsaku Tasaka, Naofumi Nomura, Mai Okazaki, Hiroyuki Shikuma, Tomoya Hatayama, Kyosuke Iwane, Ryo Tasaka, Yuki Kohada, Kenshiro Takemoto, Miki Naito, Kohei Kobatake, Yohei Sekino, Hiroyuki Kitano, Akihiro Goriki, Keisuke Hieda, Nobuyuki Hinata

**Affiliations:** ^1^ Department of Urology, Graduate School of Biomedical and Health Sciences Hiroshima University Hiroshima Japan

**Keywords:** clear cell renal cell carcinoma, external validation, Japanese cohort, lymphovascular invasion, prognostic model

## Abstract

**Objectives:**

To characterize contemporary postoperative outcomes and risk stratification in a large Japanese real‐world cohort of patients with clinically nonmetastatic clear cell renal cell carcinoma (ccRCC), and to externally validate commonly used prognostic models.

**Methods:**

We retrospectively analyzed 1677 Japanese patients with clinically nonmetastatic ccRCC who underwent radical or partial nephrectomy between 2010 and 2025. Disease‐free survival (DFS) and overall survival (OS) were evaluated. The Leibovich score, SSIGN score, AUA risk groups, and GRANT score were externally validated. Model discrimination was assessed using Harrell's C‐index.

**Results:**

During a median follow‐up of 36 months, 160 patients (9.5%) experienced recurrence and 115 (6.9%) died, including 28 renal cancer‐specific deaths. The 3‐, 5‐, and 10‐year DFS rates were 91.2%, 87.6%, and 75.9%, respectively; corresponding OS rates were 95.0%, 91.1%, and 80.9%. Pathological T stage, WHO/ISUP grade, and lymphovascular invasion (LVI) were independently associated with DFS, whereas age, ASA physical status, and LVI were independently associated with OS. All four models significantly stratified DFS and OS. For DFS, the Leibovich score showed favorable discrimination, with a C‐index of 0.785 as a continuous score and 0.769 as a risk‐group model. In a post hoc exploratory analysis, adding LVI to the Leibovich score slightly increased the C‐index for DFS, but the improvement was not statistically significant.

**Conclusions:**

Established prognostic models consistently stratified recurrence risk in Japanese patients with clinically nonmetastatic ccRCC. The Leibovich score may serve as a useful reference for postoperative risk assessment, whereas the added value of LVI requires further validation.

## 1 Introduction

Renal cell carcinoma (RCC) is the most common primary malignant tumor of the kidney, accounting for approximately 90% of all renal cancers. Worldwide, approximately 430 000 new cases and 170 000 deaths are reported annually; both incidence and mortality rates have been increasing in recent years [1]. Among its various subtypes, clear cell RCC (ccRCC) is the most prevalent, accounting for approximately 70%–75% of cases [2]. Radical nephrectomy (RN) or partial nephrectomy (PN) remains the standard treatment for localized RCC, and complete pathological resection offers the potential for long‐term survival. However, disease recurrence within a few years occurs in approximately 20%–40% of patients following radical resection [3].

The clinical landscape of postoperative management has evolved with evidence supporting the use of adjuvant immune checkpoint inhibitor (ICI) therapy in selected patients. Pembrolizumab significantly prolonged disease‐free survival (DFS) and overall survival (OS) compared with placebo in the KEYNOTE‐564 trial [4]. While eligibility criteria for adjuvant therapy are established, substantial heterogeneity remains within eligible populations: some patients face a very high risk of recurrence, whereas others may show relatively favorable outcomes even without additional treatment. Because ICI therapy is associated with immune‐related adverse events and substantial medical costs [5], postoperative decision‐making should consider not only eligibility criteria but also the risk gradient within eligible populations, together with age, comorbidities, and functional status.

Several prognostic models stratify postoperative risk in RCC, including integrated staging systems and scoring approaches based on pathological features and clinical parameters [6, 7, 8, 9]. Representative postoperative prognostic models are summarized in Table S1. The Mayo Clinic SSIGN score stratifies prognosis using stage, size, grade, and necrosis [10]. The AUA risk groups classify patients mainly by tumor stage and grade to guide postoperative surveillance [11, 12]. The GRANT score provides a simplified risk estimate using a small set of clinical variables [13]. The Leibovich score was developed to predict recurrence risk in nonmetastatic ccRCC, showing favorable predictive performance in internal and external validations [14, 15]. A revised version incorporating histological subtype was proposed in 2018, providing a modest improvement in predictive accuracy, but the model was complex [16]. Nevertheless, robust external validation of commonly used postoperative prognostic models in cohorts consisting exclusively of Japanese patients remains limited [17], and a well‐characterized reference platform from a Japanese cohort is needed to support clinical decision‐making and further development of models.

In this study, we performed survival analyses in patients with clinically nonmetastatic ccRCC and externally validated four prognostic models applicable to this cohort: the 2003 Leibovich score, SSIGN score, AUA risk groups, and GRANT score. This study aimed to identify postoperative prognostic factors, evaluate the predictive performance of these models in a Japanese population, and provide a reference for clinical decision‐making. We also explored whether additional pathological factors, including LVI, may provide incremental prognostic information within existing risk‐stratification frameworks.

## 2 Methods

### 2.1 Patient Selection

This retrospective multicenter study used data from the Hiroshima Cancer Registry Project (H‐CARP), including patients from Hiroshima University and affiliated hospitals. The study was approved by the Ethics Committee of Hiroshima University, Japan (authorization number: E‐2022‐0003). Asian Japanese patients who underwent RN or PN for clinically nonmetastatic clear cell RCC between 2010 and 2025 were eligible.

Patients who received preoperative systemic therapy or postoperative adjuvant therapy had bilateral RCC or had nonclear cell histology were excluded. Data were managed using REDCap tools hosted at Hiroshima University [18, 19], as previously described [20, 21]. Finally, 1677 patients were included in the analysis.

### 2.2 Surgical Procedures

The surgical procedure and approach were selected according to tumor characteristics, renal function, institutional practice, and surgeon expertise. RN was generally performed for locally advanced tumors or tumors unsuitable for nephron‐sparing surgery, whereas PN was preferred for small renal tumors or renal function preservation. Open, laparoscopic, or robot‐assisted approaches were used as appropriate. The adrenal gland and regional lymph nodes were removed only when clinically indicated. During PN, the tumor was excised with an adequate surgical margin, followed by renorrhaphy.

### 2.3 Clinical and Pathological Characteristics

Clinical variables were obtained from electronic medical records. Pathological variables, including pathological T stage, lymph node status, tumor size, WHO/ISUP grade, surgical margin status, LVI, sarcomatoid differentiation, and tumor necrosis, were extracted from final institutional pathology reports. Pathological staging was based on the UICC TNM Classification, 7th edition, and WHO/ISUP grade was assessed according to the WHO/ISUP 2012 grading system. Tumor necrosis was defined as microscopic coagulative necrosis.

Pathological assessment was performed by board‐certified pathologists at each institution. Central pathological review was not performed; however, predefined definitions were used for key pathological variables.

Pathologically confirmed node‐positive cases were classified as pN1. Patients who underwent lymph node dissection or sampling and showed no metastasis in the examined regional lymph nodes were classified as pN0, whereas those without pathological assessment of regional lymph nodes were classified as pNx. For clinicopathological summary and prognostic analyses, pN0 and pNx cases were combined as the node‐negative/unevaluated group [22].

### 2.4 Follow‐Up

Postoperative surveillance was based on real‐world clinical practice, generally including chest and abdominal CT approximately 3 months after surgery, every 6 months for the first 5 years, and annually thereafter. Actual surveillance was adapted according to recurrence risk, renal function, comorbidities, institutional practice, and physician judgment. Follow‐up duration was calculated from surgery to the last confirmed follow‐up or death and is reported as the median, interquartile range, and range.

### 2.5 Prognostic Models and Risk Stratification

Risk stratification was performed using the Leibovich score, SSIGN score, AUA risk groups, and GRANT score according to the original reports or guideline‐based definitions. The Leibovich and SSIGN scores were evaluated both as continuous total scores and as categorical risk classifications when applicable, whereas the AUA and GRANT models were evaluated according to their predefined risk groups.

For prognostic models requiring lymph node status, pathologically confirmed node‐positive cases were assigned to the node‐positive category. pN0 and clinically node‐negative pNx cases were treated equivalently for score calculation in this study. The potential influence of this approach was considered when interpreting the results.

### 2.6 Statistical Analysis

DFS was defined as the time from surgery to radiologically detected recurrence, distant metastasis, or last follow‐up, and OS as the time to death from any cause or last confirmed follow‐up. Survival was estimated using the Kaplan–Meier method and compared using the log‐rank test. Prognostic factors for DFS and OS were evaluated using univariate and multivariate Cox proportional hazards models. Clinically relevant variables or those significant in univariate analysis were included in multivariate models. Model discrimination was assessed using Harrell's C‐index. Differences in C‐index were estimated using the Uno method with 1000 bootstrap resamples, and multiple comparisons in the primary DFS analysis were adjusted using the Holm method. Missing data were not imputed. All analyses were performed using R software, with two‐sided *p* < 0.05 considered significant.

## 3 Results

Table 1 summarizes the clinical and pathological characteristics of the 1677 patients with clinically nonmetastatic postoperative ccRCC. The median age was 68 years (interquartile range [IQR], 59–75), and the cohort included 1196 men and 481 women. RN and PN were performed in 818 and 859 patients, respectively. The median follow‐up duration was 36 months (IQR, 17–61; range, 1–168). During follow‐up, 160 patients (9.5%) experienced recurrence, and 115 patients (6.9%) died, with 28 deaths attributable to renal cancer. Kaplan–Meier survival analysis of the entire cohort showed 2‐, 3‐, 5‐, and 10‐year DFS rates of 93.4%, 91.2%, 87.6%, and 75.9%, respectively. The corresponding OS rates were 96.9%, 95.0%, 91.1%, and 80.9%, respectively (Figure S1). Because the number of patients at risk decreased over time, particularly beyond 5 years, the 10‐year survival estimates should be interpreted cautiously.

**TABLE 1:** Patient characteristics.

			No. (%)
			*n* = 1677
Clinical	Follow up duration	Median [IQR], range, month	36 [17–61], 1–168
	Age	Median [IQR], range, years	68 [59–75], 22–93
	Sex	Female	481 (28.7)
		Male	1196 (71.3)
	Race	Japanese	1677 (100)
	ASA PS	1	290 (17.3)
		2	1107 (66)
		3	220 (13.1)
		Missing	60 (3.6)
	Location	Right	827 (49.3)
		Left	850 (50.7)
	BMI	Median[IQR], range, kg/m^2^	23.8 [21.4–26.3], 13.6–45.3
	Clinical tumor classification	T1a	1011 (60.0)
		T1b	436 (26.0)
		T2	89 (5.3)
		T3a	120 (7.2)
		T3b	14 (0.8)
		T3c	1 (0.1)
		T4	6 (0.4)
Surgical	Surgical procedure	Partial	859 (51.2)
		Radical	818 (48.8)
	Surgical approach	Robotic‐assisted	331 (19.7)
		Laparoscopic‐assisted	1253 (74.7)
		Open	93 (5.5)
Pathologic	Surgical margins	Negative	1667 (99.4)
		Positive	8 (0.5)
		Missing	2 (0.1)
	WHO/ISUP Grade	1	408 (24.3)
		2	1079 (64.3)
		3	165 (9.8)
		4	25 (1.5)
	Necrosis	No	1497 (89.3)
		Yes	178 (10.6)
		Missing	2 (0.1)
	Sarcomatoid	No	1656 (98.7)
		Yes	17 (1.0)
		Missing	4 (0.2)
	Pathologic tumor classification	T1a	1078 (64.3)
		T1b	332 (19.8)
		T2	61 (3.6)
		T3a	185 (11)
		T3b	14 (0.8)
		T3c	1 (0.1)
		T4	6 (0.4)
	Tumor size	Median [IQR], range, mm	30 [20–45], 5.7–170
	Lymphovascular Invasion	Negative	1372 (81.8)
		Positive	305 (18.2)
	Nodal involvement	Nx/N0	1667 (99.4)
		N1	10 (0.6)

Abbreviations: ASA PS, American Society of Anesthesiologists Physical Status; IQR, interquartile range.

Univariate analysis for DFS (Table 2) demonstrated that advanced age, RN, advanced pathological T stage, higher WHO/ISUP grade, larger tumor size, LVI, tumor necrosis, and sarcomatoid differentiation were significantly associated with worse DFS (all *p* < 0.05). Resection margin status and ASA Physical Status (ASA‐PS) were not significantly associated with DFS. Multivariable analysis identified pathological T stage, WHO/ISUP grade, and LVI as independent predictors of DFS. For OS (Table 3), univariate analysis showed that advanced age, higher ASA‐PS, RN, advanced pathological T stage, higher WHO/ISUP grade, larger tumor size, LVI, tumor necrosis, and sarcomatoid differentiation were significantly associated with reduced survival (all *p* < 0.05). Resection margin status was not significantly associated with OS. Multivariable analysis identified age, ASA‐PS, and LVI as independent predictors of OS.

**TABLE 2:** Univariate and multivariable analysis of DFS.

		Univariate analysis	*p*	Multivariable analysis	*p*
		HR (95% CI)		HR (95% CI)	
Age	< 68	1 (Ref.)	< 0.001	1 (Ref.)	0.087
	> 68	1.76 (1.28–2.42)		1.35 (0.96–1.90)	
ASA PS	0,1,2	1 (Ref.)	0.874	1 (Ref.)	0.71
	3	0.96 (0.59–1.57)		0.91 (0.54–1.52)	
Surgical approach	partial	1 (Ref.)	< 0.001	1 (Ref.)	0.166
	radical	2.60 (1.83–3.69)		1.33 (0.89–1.99)	
Pathological T stage	T1,2	1 (Ref.)	< 0.001	1 (Ref.)	0.002
	T3,4	5.78 (4.22–7.94)		2.17 (1.34–3.52)	
Size	< 10 cm	1 (Ref.)	< 0.001	1 (Ref.)	0.119
	> 10 cm	6.89 (4.10–11.57)		1.61 (0.88–2.94)	
Pathological N stage	N0	1 (Ref.)	0.003	1 (Ref.)	0.12
	N1	5.55 (1.76–17.47)		2.79 (0.77–10.19)	
WHO/ISUP grade	G1,2	1 (Ref.)	< 0.001	1 (Ref.)	< 0.001
	G3,4	5.46 (3.95–7.58)		2.62 (1.76–3.92)	
Surgical margins	Negative	1 (Ref.)	0.504	1 (Ref.)	0.165
	Positive	1.95 (0.273–13.97)		4.07 (0.56–29.49)	
Lymphovascular Invasion	Negative	1 (Ref.)	< 0.001	1 (Ref.)	0.006
	Positive	5.04 (3.69–6.88)		1.90 (1.20–3.00)	
Sarcomatoid change	Negative	1 (Ref.)	< 0.001	1 (Ref.)	0.566
	Positive	6.61 (2.91–14.97)		0.73 (0.25–2.15)	
Necrosis	Negative	1 (Ref.)	< 0.001	1 (Ref.)	0.142
	Positive	3.78 (2.68–5.32)		1.38 (0.90–2.11)	

Abbreviations: CI, confidence interval; HR, hazard ratio.

**TABLE 3:** Univariate and multivariable analysis of OS.

		Univariate analysis	*p*	Multivariable analysis	*p*
		HR (95% CI)		HR (95% CI)	
Age	< 68	1 (Ref.)	< 0.001	1 (Ref.)	0.001
	> 68	2.29 (1.56–3.36)		1.97 (1.31–2.97)	
ASA PS	0,1,2	1 (Ref.)	< 0.001	1 (Ref.)	< 0.001
	3	3.30 (2.15–5.05)		3.29 (2.11–5.14)	
Surgical approach	Partial	1 (Ref.)	0.003	1 (Ref.)	0.26
	Radical	1.85 (1.24–2.76)		1.30 (0.83–2.03)	
Pathological T stage	T1,2	1 (Ref.)	< 0.001	1 (Ref.)	0.75
	T3,4	2.61 (1.72–3.95)		1.10 (0.60–2.02)	
Size	< 10 cm	1 (Ref.)	0.008	1 (Ref.)	0.41
	> 10 cm	2.65 (1.29–5.45)		1.44 (0.61–3.43)	
Pathological N stage	N0	1 (Ref.)	0.039	1 (Ref.)	0.15
	N1	4.41 (1.08–17.97)		3.09 (0.66–14.55)	
WHO/ISUP grade	G1,2	1 (Ref.)	< 0.001	1 (Ref.)	0.16
	G3,4	2.55 (1.65–3.95)		1.47 (0.85–2.54)	
Surgical margins	Negative	1 (Ref.)	0.28	1 (Ref.)	0.09
	Positive	2.97 (0.41–21.35)		5.60 (0.77–41.02)	
Lymphovascular Invasion	Negative	1 (Ref.)	< 0.001	1 (Ref.)	0.04
	Positive	2.32 (1.57–3.44)		1.79 (1.03–3.10)	
Sarcomatoid change	Negative	1 (Ref.)	< 0.001	1 (Ref.)	0.38
	Positive	4.87 (1.96–12.07)		1.73 (0.51–5.93)	
Necrosis	Negative	1 (Ref.)	< 0.001	1 (Ref.)	0.55
	Positive	2.22 (1.41–3.49)		1.19 (0.67–2.12)	

Abbreviations: CI, confidence interval; HR, hazard ratio.

**FIGURE 1:**
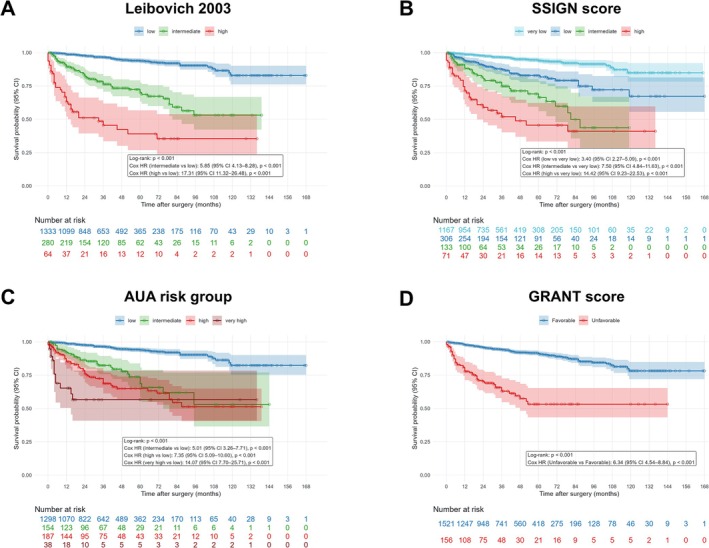
Kaplan–Meier curves for disease‐free survival (DFS) stratified by established prognostic models. Kaplan–Meier curves for DFS stratified by (A) Leibovich score (2003), (B) SSIGN score, (C) AUA risk classification, and (D) GRANT score. All four models significantly stratified postoperative DFS, with clear separation of survival curves among risk groups (log‐rank *p* < 0.001 for all models). Multicategory models showed stepwise deterioration in prognosis, whereas the binary GRANT score demonstrated a clear survival difference.

**FIGURE 2:**
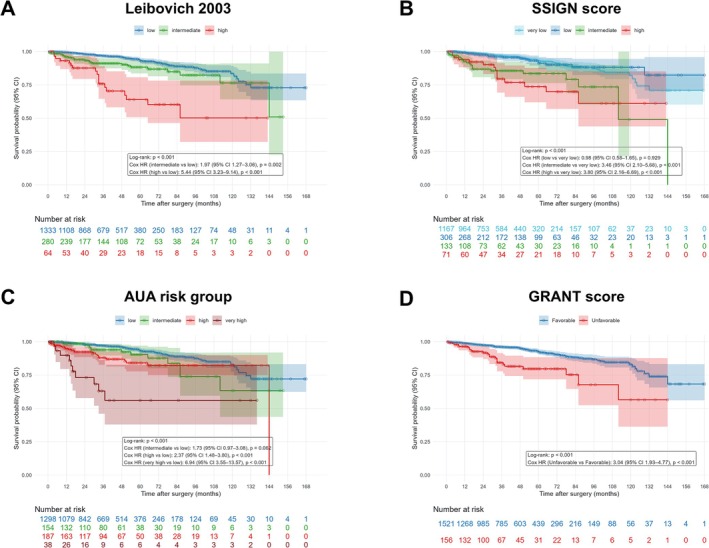
Kaplan–Meier curves for overall survival (OS) stratified by established prognostic models: Kaplan–Meier curves for OS stratified by (A) Leibovich score (2003), (B) SSIGN score, (C) AUA risk classification, and (D) GRANT score. All models demonstrated significant OS stratification, with trends similar to those observed for disease‐free survival (log‐rank *p* < 0.001 for all models).

Patients were stratified using the Leibovich score, SSIGN score, AUA risk groups, and GRANT score. Kaplan–Meier curves for DFS are shown in Figure 1. All four models demonstrated significant stratification of postoperative DFS in this external validation cohort, with clear separation of survival curves between risk groups (log‐rank *p* < 0.001 for all models). Multicategory models, including the Leibovich score, SSIGN score, and AUA risk groups, showed stepwise deterioration in prognosis, whereas the binary GRANT score demonstrated a clear survival difference. Similar findings were observed for OS (Figure 2).

Discriminatory performance was assessed using Harrell's C‐index (Table 4). Because the Leibovich and SSIGN scores can be evaluated either as continuous total scores or as categorical risk classifications, these approaches were assessed separately. When evaluated as continuous scores, the C‐index for DFS was 0.785 (95% CI, 0.744–0.827) for the Leibovich score and 0.773 (95% CI, 0.733–0.814) for the SSIGN score, with no significant difference between the two. The corresponding C‐index values for OS were 0.655 (95% CI, 0.594–0.716) for the Leibovich score and 0.655 (95% CI, 0.595–0.715) for the SSIGN score.

**TABLE 4:** Discriminatory performance of prognostic models for DFS and OS.

Endpoint	Model	Format	c‐index	95% CI
DFS	Leibovich score 2003	Continuous score	0.785	0.744–0.827
DFS	Leibovich score 2003	Risk group	0.769	0.729–0.808
DFS	SSIGN score	Continuous score	0.773	0.733–0.814
DFS	AUA group	Risk group	0.758	0.718–0.798
DFS	GRANT score	Risk group	0.662	0.621–0.704
OS	Leibovich score 2003	Continuous score	0.655	0.594–0.716
OS	Leibovich score 2003	Risk group	0.653	0.598–0.707
OS	SSIGN score	Continuous score	0.655	0.595–0.715
OS	AUA group	Risk group	0.658	0.601–0.714
OS	GRANT score	Risk group	0.583	0.537–0.629

*Note:* The Leibovich score was evaluated both as a continuous total score and as a categorical risk‐group model. AUA and GRANT models were evaluated according to their predefined risk groups.

Abbreviations: CI, confidence interval; C‐index, concordance index; DFS, disease‐free survival; OS, overall survival.

When evaluated as group‐based models, the C‐index values for DFS were 0.769 (95% CI, 0.729–0.808), 0.758 (95% CI, 0.718–0.798), and 0.662 (95% CI, 0.621–0.704) for the Leibovich score, AUA risk groups, and GRANT score, respectively. Bootstrap analysis showed significantly higher discriminatory ability for the Leibovich and AUA risk groups than for the GRANT score (both *p* < 0.001), with no significant difference between the Leibovich and AUA risk groups. Similar trends were observed for OS, with both the Leibovich and AUA risk groups outperforming the GRANT score (*p* < 0.02).

Overall, the Leibovich score demonstrated robust risk stratification and favorable discriminatory performance, particularly for DFS.

Because LVI was independently associated with both DFS and OS, we performed a post hoc exploratory analysis to assess whether LVI could provide additional prognostic information within the established Leibovich‐based framework. LVI was assigned 2 points, yielding a total score range of 0 to 12 points, and patients were classified into low‐ (0–2 points), intermediate‐ (3–6 points), and high‐risk (≥ 7 points) groups.

**FIGURE 3:**
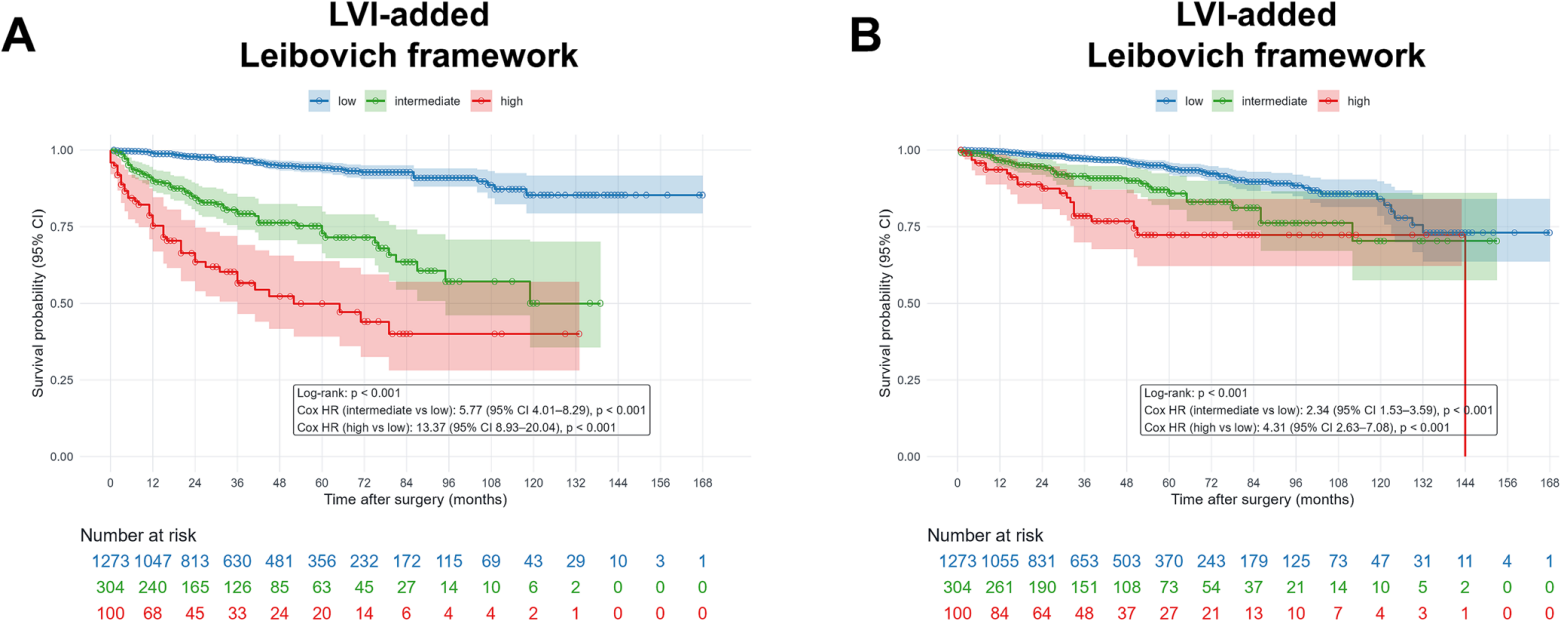
Kaplan–Meier curves according to the LVI‐added Leibovich framework. Kaplan–Meier curves for disease‐free survival (DFS) (A) and overall survival (OS) (B) stratified by the LVI‐added Leibovich framework. LVI was assigned 2 points and added to the original Leibovich score, yielding a total score range of 0–12 points. Patients were classified into low‐risk (0–2 points), intermediate‐risk (3–6 points), and high‐risk (≥ 7 points) groups. The shaded areas indicate 95% confidence intervals, and the numbers at risk are shown below each plot. Log‐rank tests and Cox proportional hazards models were used to compare survival among groups.

The LVI‐added Leibovich framework showed stepwise separation of DFS and OS among risk groups (Figure 3). For DFS, the HRs for the intermediate‐ and high‐risk groups compared with the low‐risk group were 5.77 (95% CI, 4.01–8.29; *p* < 0.001) and 13.37 (95% CI, 8.93–20.04; *p* < 0.001), respectively. For OS, the corresponding HRs were 2.34 (95% CI, 1.53–3.59; *p* < 0.001) and 4.31 (95% CI, 2.63–7.08; *p* < 0.001), respectively.

Although the LVI‐added Leibovich framework showed slightly higher C‐index values than the original Leibovich score for DFS (0.796 vs. 0.785) and OS (0.657 vs. 0.655), these differences were not statistically significant. Therefore, the incremental discriminatory value of adding LVI was limited in this cohort.

## 4 Discussion

In this large real‐world cohort of 1677 Japanese patients with clinically nonmetastatic ccRCC, we externally validated representative postoperative prognostic models and characterized contemporary outcomes. All evaluated models, including the Leibovich score, SSIGN score, AUA risk groups, and GRANT score, significantly stratified postoperative recurrence risk. Among them, the Leibovich score showed robust discriminatory performance for DFS and performed comparably to or better than other established models. These findings suggest that prognostic models developed mainly in Western populations can also provide clinically useful postoperative risk stratification in Japanese patients.

External validation studies of postoperative prognostic models in Japanese patients remain limited [23, 24]. Although selected models, such as the SSIGN score and several nomograms, have been evaluated previously, few studies have compared multiple representative models in a contemporary Japanese real‐world cohort. In this study, the Leibovich and SSIGN scores showed favorable discrimination for recurrence‐related outcomes, whereas the GRANT score showed relatively lower discrimination. This may reflect the trade‐off between simplicity and granularity, as models incorporating fewer pathological variables may be easier to apply but less precise for recurrence risk stratification.

All evaluated models showed lower discriminatory performance for OS than for DFS. This is clinically plausible because OS is influenced not only by tumor biology but also by age, performance status, comorbidities, noncancer‐related mortality, and treatment after recurrence [25]. In this cohort, only 28 deaths were attributed to renal cancer, indicating that noncancer‐related deaths accounted for a substantial proportion of OS events. Although cancer‐specific survival or competing‐risk analyses would be useful for evaluating tumor‐specific outcomes [26], the small number of cancer‐specific deaths limited the reliability of such analyses. Therefore, OS findings should be interpreted as complementary to DFS rather than as direct measures of cancer‐specific prognosis.

The lower C‐index values for OS may also reflect heterogeneity in postrecurrence management and temporal changes in systemic therapy. This cohort included patients treated between 2010 and 2025, during which systemic treatment for advanced RCC changed substantially with the introduction of immune checkpoint inhibitor‐based regimens. Thus, OS may have been affected by subsequent treatment availability, treatment era, comorbidities, and noncancer‐related mortality. In contrast, DFS may be a more direct endpoint for evaluating postoperative recurrence risk models.

Another important consideration is that the evaluated models were originally developed using different endpoints, including recurrence, progression, cancer‐specific survival, and overall survival. Therefore, direct comparison using uniform endpoints of DFS and OS may not fully reflect each model's original intended use. This should be considered when interpreting differences in C‐index among models.

Beyond validating existing tools, our findings underscore the clinical relevance of LVI as a marker of tumor aggressiveness. LVI was independently associated with both DFS and OS, supporting its role as a clinically meaningful pathological factor. We therefore performed a post hoc exploratory analysis to assess whether LVI could provide additional prognostic information within the established Leibovich‐based framework. Although the LVI‐added Leibovich framework showed numerically higher C‐index values than the original Leibovich score, the differences were small and not statistically significant. Thus, this framework should not be regarded as a validated new prognostic model, but rather as an exploratory analysis suggesting that LVI may be considered in future model‐refinement studies.

From a contemporary clinical perspective, postoperative prognostication has become increasingly important in the era of adjuvant immune checkpoint inhibitor therapy. Although eligibility criteria for adjuvant therapy have been defined, recurrence risk remains heterogeneous even among patients meeting conventional high‐risk criteria. Refined risk stratification may therefore support shared decision‐making regarding adjuvant therapy and surveillance intensity, particularly when considering immune‐related adverse events, medical costs, age, comorbidities, and functional status [27, 28].

Patients with very early postoperative recurrence may have had occult metastatic disease at the time of nephrectomy and may represent a biologically distinct subgroup. Future studies should evaluate whether established prognostic models can identify such patients and whether integration of pathological, radiological, and molecular markers can further improve risk stratification.

This study has several limitations. First, the retrospective design may have introduced selection bias and unmeasured confounding. Second, this multicenter real‐world cohort was collected over a long period, and institutional or temporal variability may have influenced surveillance, outcome assessment, and risk estimation. Third, central pathological review was not performed, which may have affected the consistency of pathological assessment. However, pathological variables were extracted from final pathology reports using predefined definitions. Fourth, lymph node status should be interpreted with caution. Because lymph node dissection was not routinely performed in clinically node‐negative patients, complete distinction between pN0 and pNx was difficult in the original database. Although pN1 cases were reliably identified and pN0/clinically node‐negative pNx cases were treated equivalently for score calculation, this limitation may have influenced risk‐score assignment. Fifth, the limited number of outcome events and modest follow‐up duration may have affected the stability of multivariable analyses and long‐term survival estimates, particularly for OS. Finally, the LVI‐added Leibovich framework was evaluated only as a post hoc exploratory analysis and requires external validation before clinical application.

In conclusion, established prognostic models consistently stratified recurrence risk in this Japanese real‐world cohort of clinically nonmetastatic ccRCC. The Leibovich score showed robust DFS performance and may serve as a useful reference for postoperative risk assessment. Although LVI was independently associated with outcomes, its added value was limited and should be interpreted as exploratory. Further studies with longer follow‐up and external validation are needed.

## Author Contributions

**Kazuma Yukihiro:** investigation. **Yoshinori Nakano:** conceptualization, investigation, methodology, data curation, formal analysis, validation, software, writing – original draft, visualization. **Mai Okazaki:** investigation. **Shinsaku Tasaka:** investigation. **Naofumi Nomura:** investigation. **Hiroyuki Shikuma:** investigation. **Tomoya Hatayama:** investigation. **Shunsuke Miyamoto:** conceptualization, data curation, project administration, methodology, writing – review and editing. **Kyosuke Iwane:** investigation. **Ryo Tasaka:** investigation. **Yuki Kohada:** investigation. **Kenshiro Takemoto:** investigation. **Miki Naito:** investigation. **Keisuke Goto:** investigation, methodology. **Kohei Kobatake:** investigation. **Yohei Sekino:** investigation. **Hiroyuki Kitano:** investigation. **Akihiro Goriki:** investigation. **Keisuke Hieda:** investigation. **Nobuyuki Hinata:** project administration, supervision.

## Funding

The authors have nothing to report.

## Ethics Statement

This study was approved by the Institutional Review Board of Hiroshima University Hospital (approval no. E‐2022‐0003). All procedures were conducted in accordance with the Declaration of Helsinki.

## Consent

Informed consent was waived due to the retrospective nature of the study and the use of anonymized data, in accordance with the regulations of the Institutional Review Board of Hiroshima University.

## Conflicts of Interest

Nobuyuki Hinata is an Editorial Board member of International Journal of Urology and a coauthor of this article. To minimize bias, they were excluded from all editorial decision‐making related to the acceptance of this article for publication.

## Supporting information


**Figure S1:** Kaplan–Meier survival curves for the entire cohort. Kaplan–Meier curves showing (A) disease‐free survival (DFS) and (B) overall survival (OS) for the entire cohort of patients with nonmetastatic renal cell carcinoma after surgery. The 2‐, 3‐, 5‐, and 10‐year DFS rates were 93.4%, 91.2%, 87.6%, and 75.9%, respectively, while the corresponding OS rates were 96.9%, 95.0%, 91.1%, and 80.9%.

**Table S1:** Summary of representative postoperative prognostic models/nomograms for renal cell carcinoma (RCC), including endpoints, study populations, variables incorporated, and scoring/risk stratification methods. Representative prognostic models/nomograms for renal cell carcinoma (RCC) after nephrectomy. The table summarizes each model's original report (author/year), endpoint(s), study population, included variables, and scoring system/risk stratification approach. AUA, American Urological Association; ccRCC, clear cell renal cell carcinoma; chrRCC, chromophobe renal cell carcinoma; CSS, cancer‐specific survival; DFS, disease‐free survival; ECOG, Eastern Cooperative Oncology Group; OS, overall survival; papRCC, papillary renal cell carcinoma; PFS, progression‐free survival; RCC, renal cell carcinoma; RFS, recurrence‐free survival; SSIGN, Stage, Size, Grade, and Necrosis; UISS, UCLA Integrated Staging System.

## Data Availability

The data that support the findings of this study are available from the corresponding author upon reasonable request.
