# Supplementary figures and images for: External Validation of Prognostic Models for Nonmetastatic Clear Cell Renal Cell Carcinoma in a Japanese Cohort: Evaluation of Predictive Performance
Source: Int J Urol. 2026 Jul 23;33(7):e70579. doi: 10.1111/iju.70579 (PMC13392575; doi:10.1111/iju.70579)

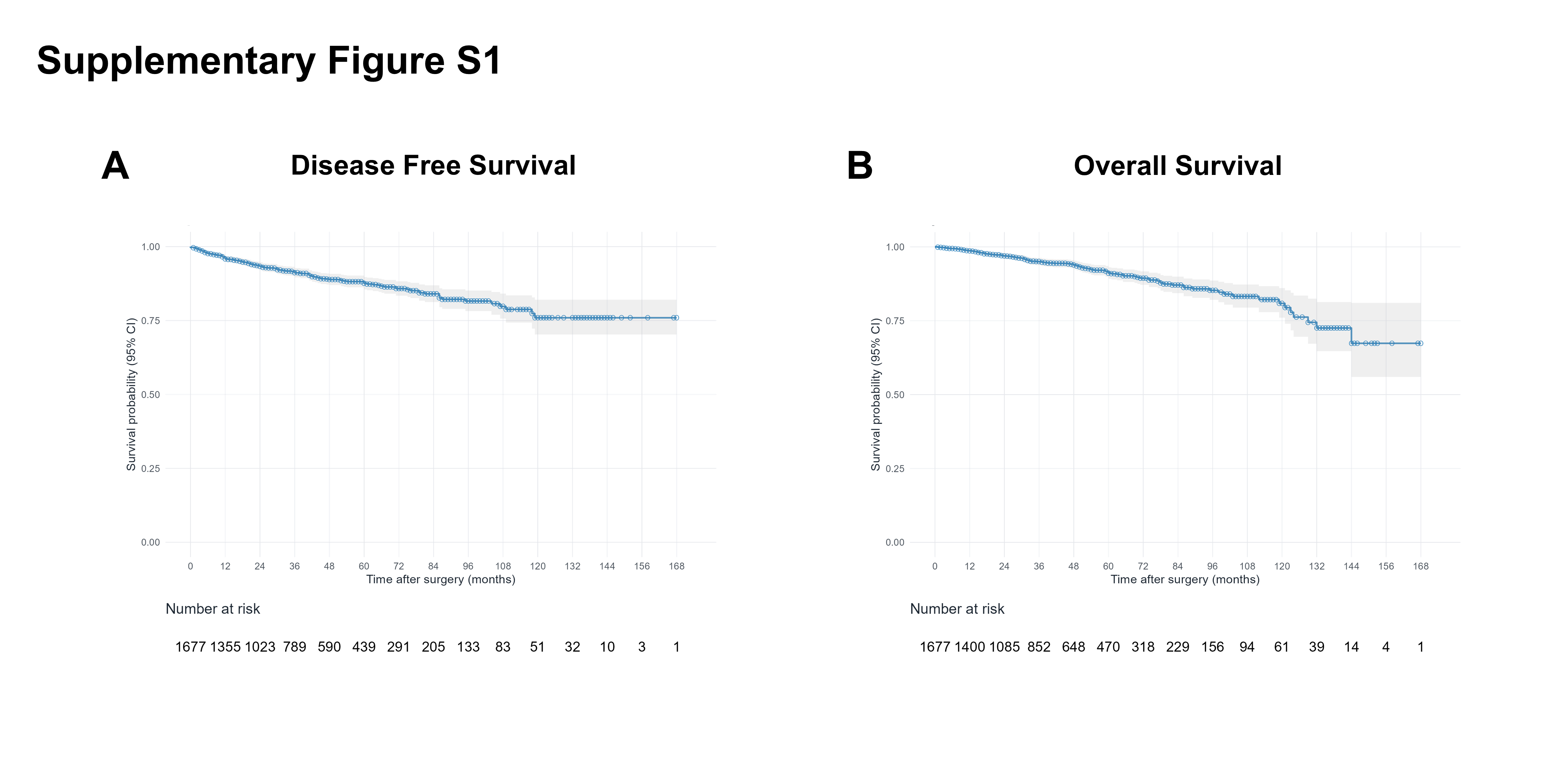

Supplement: Supplementary file 1 — Figure S1: Kaplan–Meier survival curves for the entire cohort. Kaplan–Meier curves showing (A) disease‐free survival (DFS) and (B) overall survival (OS) for the entire cohort of patients with nonmetastatic renal cell carcinoma after surgery. The 2‐, 3‐, 5‐, and 10‐year DFS rates were 93.4%, 91.2%, 87.6%, and 75.9%, respectively, while the corresponding OS rates were 96.9%, 95.0%, 91.1%, and 80.9%. [file IJU-33-0-s001.tif]
