# Supplementary material for: External Validation of Prognostic Models for Nonmetastatic Clear Cell Renal Cell Carcinoma in a Japanese Cohort: Evaluation of Predictive Performance
Source: Int J Urol. 2026 Jul 23;33(7):e70579. doi: 10.1111/iju.70579 (PMC13392575; doi:10.1111/iju.70579)
Supplement: Supplementary file 2 — Table S1: Summary of representative postoperative prognostic models/nomograms for renal cell carcinoma (RCC), including endpoints, study populations, variables incorporated, and scoring/risk stratification methods. Representative prognostic models/nomograms for renal cell carcinoma (RCC) after nephrectomy. The table summarizes each model's original report (author/year), endpoint(s), study population, included variables, and scoring system/risk stratification approach. AUA, American Urological Association; ccRCC, clear cell renal cell carcinoma; chrRCC, chromophobe renal cell carcinoma; CSS, cancer‐specific survival; DFS, disease‐free survival; ECOG, Eastern Cooperative Oncology Group; OS, overall survival; papRCC, papillary renal cell carcinoma; PFS, progression‐free survival; RCC, renal cell carcinoma; RFS, recurrence‐free survival; SSIGN, Stage, Size, Grade, and Necrosis; UISS, UCLA Integrated Staging System. [file IJU-33-0-s002.docx]

Supplementary table S1

| **Model** | **Author/Year** | **Endpoint(s)** | **Study population** | **Variables included** | **Scoring / risk stratification** |
| --- | --- | --- | --- | --- | --- |
| UISS | Amnon Zisman, 2001 [6] | OS / RFS | RCC patients (all histological subtypes) undergoing nephrectomy | TNM stage, Fuhrman grade, ECOG performance status | Stratified into five risk groups |
| Kattan nomogram | M W Kattan, 2001 [7] | RFS | RCC patients (all histological subtypes) undergoing nephrectomy | Presence of symptoms, histology, tumor size, pathological stage | Nomogram estimating 5-year recurrence-free survival |
| Cindolo preoperative model | L Cindolo, 2003 [9] | DFS | Non-metastatic RCC patients assessed preoperatively | Presence of clinical symptoms, clinical tumor size | Two risk groups using the Recurrence Risk Formula |
| SSIGN | Frank I, 2002 [10] | CSS / DFS | ccRCC patients undergoing radical nephrectomy | Pathological stage, tumor size, Fuhrman grade, tumor necrosis | Risk stratification based on total score |
| Leibovich score (2003) | Leibovich BC, 2003 [14] | DFS / CSS | Non-metastatic ccRCC patients undergoing radical nephrectomy | T stage, N stage, tumor size, grade, necrosis | Three risk groups based on total score |
| AUA risk groups | Donat SM, 2013 [11] | PFS / CSS | Non-metastatic ccRCC and papRCC after surgery | T stage, grade, N stage, sarcomatoid/rhabdoid features, surgical margin status | Stratified into four risk groups |
|  | Steven C, 2021 [12] |  |  |  |  |
| Yaycioglu preoperative nomogram | Yaycioglu O, 2013 [8] | OS / RFS | Non-metastatic RCC patients evaluated preoperatively | Presence of symptoms, tumor size (imaging-based) | Two risk groups based on total score |
| GRANT score | Buti S, 2017 [13] | DFS / OS | ccRCC and non-ccRCC patients undergoing nephrectomy | T stage, N stage, age, grade | Two risk groups based on total score |
| Leibovich score (2018) | Leibovich BC, 2018 [16] | PFS / CSS | Non-metastatic ccRCC,papRCC,chrRCC patients undergoing radical nephrectomy | Constitutional symptoms, grade, necrosis, sarcomatoid differentiation. tumor size, perinephric or renal sinus fat invasion, tumor thrombus level, extension beyond kidney, nodal involvement | Risk stratification based on total score |
|  | ccRCC; clear cell renal cell carcinoma, papRCC; papillary renal cell carcinoma, chrRCC; chromophobe renal cell carcinoma | | | | |
